# Gene expression and proteomic analysis of the formation of *Phakopsora pachyrhizi* appressoria

**DOI:** 10.1186/1471-2164-13-269

**Published:** 2012-06-22

**Authors:** Christine L Stone, Michael B McMahon, Laurie L Fortis, Alberto Nuñez, Gary W Smythers, Douglas G Luster, Reid D Frederick

**Affiliations:** 1 USDA-Agricultural Research Service, Foreign Disease-Weed Science Research Unit, 1301 Ditto Avenue, Fort Detrick, MD, 21702, USA; 2 USDA-Agricultural Research Service, Eastern Regional Research Center, 600 East Mermaid Lane, Wyndmoor, PA, 19038, USA; 3 National Cancer Institute, Advanced Biomedical Computing Center, Building 430, Fort Detrick, MD, 21702, USA; 4 Present address: USDA-National Institute of Food and Agriculture, Institute of Bioenergy, Climate, and Environment, 3245 Waterfront Centre, 800 9th Street, Southwest, Washington, District of Columbia, 20024, USA

## Abstract

**Background:**

*Phakopsora pachyrhizi* is an obligate fungal pathogen causing Asian soybean rust (ASR). A dual approach was taken to examine the molecular and biochemical processes occurring during the development of appressoria, specialized infection structures by which *P. pachyrhizi* invades a host plant. Suppression subtractive hybridization (SSH) was utilized to generate a cDNA library enriched for transcripts expressed during appressoria formation. Two-dimensional gel electrophoresis and mass spectroscopy analysis were used to generate a partial proteome of proteins present during appressoria formation.

**Results:**

Sequence analysis of 1133 expressed sequence tags (ESTs) revealed 238 non-redundant ESTs, of which 53% had putative identities assigned. Twenty-nine of the non-redundant ESTs were found to be specific to the appressoria-enriched cDNA library, and did not occur in a previously constructed germinated urediniospore cDNA library. Analysis of proteins against a custom database of the appressoria-enriched ESTs plus Basidiomycota EST sequences available from NCBI revealed 256 proteins. Fifty-nine of these proteins were not previously identified in a partial proteome of *P. pachyrhizi* germinated urediniospores. Genes and proteins identified fell into functional categories of metabolism, cell cycle and DNA processing, protein fate, cellular transport, cellular communication and signal transduction, and cell rescue. However, 38% of ESTs and 24% of proteins matched only to hypothetical proteins of unknown function, or showed no similarity to sequences in the current NCBI database. Three novel *Phakopsora* genes were identified from the cDNA library along with six potentially rust-specific genes. Protein analysis revealed eight proteins of unknown function, which possessed classic secretion signals. Two of the extracellular proteins are reported as potential effector proteins.

**Conclusions:**

Several genes and proteins were identified that are expressed in *P*. *pachyrhizi* during appressoria formation. Understanding the role that these genes and proteins play in the molecular and biochemical processes in the infection process may provide insight for developing targeted control measures and novel methods of disease management.

## Background

Asian soybean rust (ASR), caused by the fungal pathogen *Phakopsora pachyrhziri* Sydow & Sydow is an aggressive foliar pathogen of soybeans. Initially identified in Asia, it has since spread to all major soybean-growing regions of the world, including the United States [1,2]. The impact of disease on crop yields is influenced by temperature and humidity, spore load introduced into a field, and the growth stage of soybeans when first infection occurs. Field trials in Brazil found yield loss averaging 37% when infection began at R5 growth stage and 67% when infection started at R2 [3]. Observations in Asia have reported yield losses up to 80% under high disease pressure and favorable environmental conditions [4,5].

While fungicide applications are able to reduce yield loses, a limited number of fungicides are available for foliar application on soybeans. The cost of fungicide applications, efficacy of treatments on maturing plants and dense canopies, and environmental impact are all considerations for the soybean grower. Six resistance genes (*Rpp1Rpp6*) have been identified that provide resistance to *P*. *pachyrhizi*[6-11]. However, these genes display only race-specific resistance to selected isolates of *P. pachyrhizi*.

When a urediniospore makes contact with a soybean leaf, a single germ tube elongates across the leaf surface. At the tip of this germ tube a specialized infection structure, the appressorium, is formed. While most rust pathogens penetrate the host indirectly by entering through stomatal openings and then breaching the mesophyll cell wall, *P. pachyrhizi* is one of a few rusts that enter the host by direct penetration of the cuticle and epidermal cell wall [12]. Recent transmission electron microscopy confirmed that *P. pachyrhizi* uses mechanical force to penetrate the cuticle and digestive enzymes to penetrate the epidermal cell wall [13]. The fungus continues to grow invasively in the host forming haustoria, colonizing hyphae, and ultimately uredinia.

In a previous study, a cDNA library was utilized to evaluate gene expression during urediniospore germination of *P. pachyrhizi*[14]. A subsequent independent study examined the partial proteome of germinated urediniospores [15]. The next critical step in the infection cycle is appressoria formation. *P. pachyrhizi* infects over 90 species of legumes, and this broad host range is unique among the rusts [16]. Elucidating the events during appressoria formation could shed light on the mechanism allowing broad-spectrum interaction.

Obligate pathogens require a living host on which to survive and propagate, making it difficult to separate fungal-specific genes or proteins from those of the host. However, induction of appressoria by surface contact with artificial substrates is possible for some fungi, including *P. pachyrhizi*[17,18]. Despite the ability to induce appressoria formation *in vitro*, a review of the literature found appressoria-specific EST libraries generated only for the plant pathogens *Magnaporthe griseaPuccinia triticina*, and *Colletotrichum higginsianum*[19-21].

Several studies have utilized bioinformatics to identify appressoria proteins from EST sequencing projects [21,22], but only a few have used a proteomics approach to identify differentially expressed proteins accumulated during appressoria formation. For example, comparison of protein expression patterns in germinating conidia to those observed in appressoria revealed five proteins that were up-regulated during appressoria formation in *M. grisea*[23]. Protein profiles of three developmental stages of *Phytophthora infestans* (cysts, germinated cysts, and appressoria-forming cysts) found 13 proteins to be up- or down-regulated during different developmental stages [24]. Likewise, another study of *P. infestans* identified four up-regulated genes and their protein products in cysts with appressoria [25].

This study identified ESTs and proteins present during, and possibly required for, appressoria formation in *P. pachyrhizi*. This is one of the few studies to evaluate genes or proteins present specifically during appressoria formation and the first to combine the two techniques. The comparison of identified transcripts to accumulated proteins allows for a more comprehensive analysis of the molecular and biochemical processes occurring during appressoria formation.

## Methods

### Fungal strain and growth conditions

*P. pachyrhizi* isolate Taiwan 72-1 was maintained at the USDA-ARS Foreign Disease-Weed Science Research Unit Plant Pathogen Containment Facility at Fort Detrick, MD [26] under Animal and Plant Health Inspection Service permit. Urediniospores were obtained and maintained as described previously [27].

Urediniospores were germinated by floating on the surface of sterile distilled water containing 50 μg/ml each of ampicillin and streptomycin in a 9” x 13” glass baking dish, for 6 h at room temperature in the dark. Germinated urediniospores were collected onto Whatman No. 1 filter paper (Whatman; Piscataway, NJ) and flash frozen in liquid nitrogen.

Appressoria were generated by application of 150 ml of a 100,000 urediniospore/ml suspension onto a 245 mm x 245 mm x 20 mm polystyrene dish, followed by incubation in the dark at room temperature for 6 h. Water was decanted from the plates and collected for protein extraction. This collected water will subsequently be referred to as the Appressoria Water Fraction (AWF). A sterile water wash was used to remove urediniospores that did not germinate from the plate. Plates were examined under a microscope before and after washing to confirm the presence of appressoria. Appressoria, germ tubes, and germinated urediniospores were collected by scraping the plate surface with a cell scraper (Nunc, Thermo Fisher Scientific; Rochester, NY) and immediately frozen and stored in liquid nitrogen.

### RNA isolation and library construction

Germinated urediniospores and appressoria were each separately ground under liquid nitrogen with a mortar and pestle. Total RNA was isolated by phenol:chloroform extraction [28], and precipitated overnight with 12 M lithium chloride.

Purification of poly(A)^+^ mRNA, synthesis of cDNA, and library construction was performed by SeqWright (Houston, TX) following standard protocols for library construction via suppression subtractive hybridization [29]. The driver for the subtracted library was generated from 1400 μg of total RNA extracted from germinated urediniospores, and 1400 μg of total RNA from appressoria was used as the tester. The subtracted-cDNA pool was cloned into pBluescript II KS(+) vector (Stratagene; La Jolla, CA) and transformed into DH10B *E.coli* strain (Invitrogen; Carlsbad, CA). Average insert size for the library was determined by plasmid mini-prep, PCR using T3 and T7 primers, and gel electrophoresis of 15 randomly selected colonies from the library (performed by SeqWright).

### Sequence analysis

Single-pass sequencing of clones from the cDNA library was performed on an Applied Biosystems 3700 DNA analyzer (Applied Biosystems; Framingham, MA) at the USDA-ARS Eastern Regional Research Center Nucleic Acids Facility (Wyndmoor, PA). Initial sequence analysis was performed at the Advanced Biomedical Computing Center at the National Cancer Institute-Frederick (ABCC/NCI-Frederick) (Frederick, MD). Sequenced clones were subjected to BLASTN analysis against all non-chordate ESTs in the GenBank database to identify clones with similarity to existing *P. pachyrhizi* ESTs [14, http://www.ncbi.nlm.nih.gov/nucest/?term=txid170000[Organism:noexp]. Clones that did not share similarity to these *P. pachyrhizi* ESTs were subjected to additional, bi-directional sequencing, using anchored poly(T)N primers to read through poly(A)^+^ tails. Additional primers, as needed, were designed using Primer3 (http://frodo.wi.mit.edu/primer3/) [30]. Subsequent sequence analysis and compilation of full-length sequences was performed using Chromas 2.33 (Techelysium Pty; Helensvale, Australia). Putative functions were assigned to clones by BLASTX analysis against the NCBI non-redundant protein database. Putative functional categories were determined using the Munich Information Center of Protein Sequences (MIPS) Functional Catalogue (http://mips.helmholtz-muenchen.de/proj/funcatDB/search_main_frame.html).

Redundancy within the subtracted library was identified by BLASTN analysis of the library against itself. Redundant and overlapping ESTs were assembled into contigs using the program CAP3 (http://pbil.univ-lyon1.fr/cap3.php) [31].

### Quantitative real-time RT-PCR analysis of transcript levels during the infection cycle

The soybean cultivar Williams 82 was inoculated with *P. pachyrhizi* isolate Taiwan 72-1, as described previously [27]. Leaves were collected from three biological replicates at 0, 6, 12, 24, 72, 168, and 336 h post inoculation (hpi). Freshly germinated urediniospores and appressoria, separate from that used to generate the cDNA library, were produced as described above. Total RNA was isolated from 100 mg of each sample and 100 mg of urediniospores using the RNeasy Mini Plant kit (Qiagen; Valencia, CA) following the manufacturer’s protocol.

Six ESTs specific to the appressoria-enriched cDNA library and representing four putative functional categories and one unclassified protein were selected for transcript analysis. Three ESTs common to the appressoria-enriched cDNA library and the germinated urediniospore library [14] were also selected for analysis. Nucleotide sequences of each clone were compared to the Trace Archives for *P. pachyrhizi* Whole Genome Shotgun (WGS) sequences by BLASTN to identify the positions of potential introns. Primers were designed to span putative inrons where possible, using the primer design program Primer3 (http://frodo.wi.mit.edu/primer3/).

To assess transcript levels quantitative real-time RT-PCR (qRT-PCR) was performed using three biological replicates and two technical replicates for each template. First-strand cDNA synthesis was performed using the QuantiTect Reverse Transcription kit (Qiagen) following the manufacturer’s protocol. Real-time PCR reactions were performed on the SmartCycler System (Cepheid; Santa Clara, CA) using the QuantiTect SYBR Green PCR kit (Qiagen) following the manufacturer’s protocol. Sequences and annealing temperature for each primer set are listed in Table 1. Melt curve analysis was performed to verify specificity of the PCR products, and control reactions containing no template or DNA without reverse transcriptase were included. Absolute quantification of target molecules was conducted using the sigmoidal model, and lambda gDNA was used to generate the optical calibration factor (OCF) [32]. Primers for α-tubulin were included to assess RNA integrity and demonstrate functionality of the RT-PCR assay [33]. For each time point, the average number of target molecules and standard deviation was calculated using the three biological replicates. Data were analysed by analysis of variance.

**Table 1:** **Primer pairs used for real-time quantitative reverse transcription PCR analysis of expression patterns of*Phakopsora pachyrhizi*ESTs**

**Clone/contig**	**Target gene product**		**Primer sequences**	**Amplicon size (bp)**	**Primer (nM)**	**Annealing temperature (°C)**
Pp3004^1^	serine/threonine-protein kinase	Forward	GGAACCAACGTCGACAAGAG	130	200	60
		Reverse	CTGGTCCTCCTCAACCTCAG			
Pp3243^1^	prefoldin subunit 5	Forward	AACCTGATCAACTGGCATCC	81	200	59
		Reverse	CCTTTAGTTGCCCAAACGAG			
Pp3282^1^	ubiquitin-protein ligase	Forward	AAATTCCTGGAGACGATTGC	150	300	60
		Reverse	TTGCACTACCTTGTGCGGTA			
Pp3495^1^	G2/M phase checkpoint control protein Sum2	Forward	CAGCCTAGAACAGGTCAGATCC	105	200	58
		Reverse	GCCCGGAAAACGATAAATTC			
Pp3505^1^	hypothetical protein	Forward	GAGACAAGCCCCATTGAGAG	104	200	60
		Reverse	GTCTTTGGCAGGGTCTTCTG			
Pp3684^1^	HMG-CoA reductase	Forward	CGACAGCTTGCTCGAATTATC	87	200	60
		Reverse	CTTAACCAAGTGTCCAGCTGC			
contig2F^1^	5-aminolevulinate synthase	Forward	GATGAGGTTCACGCCATTG	117	200	60
		Reverse	GTCCACCCGATCCATTACAC			
Pp3186^1^	hypothetical protein	Forward	AAGGACTGGTGAGGTTGGTG	76	200	62
		Reverse	TGCTCTACCACCGTAGGACC			
contig2N^1^	P-type cation-transporting ATPase	Forward	GCCTGAAGTGATTCCTCAGC	124	400	55
		Reverse	ATCCCAACAAACGAAAGTG			
Pp3042^2^	GTPase-binding protein	Forward	TGCCCAGAAAATTGGTTCTC	102	300	58
		Reverse	GCCAAAAGTGCATACCGAGT			
Pp3205^2^	NADPH oxidase A	Forward	ACCCAAGGCTGCCATTAGTA	134	300	56
		Reverse	GGTGCCCTCCATAAAACAAA			
Pp3222^2^	septin/cell division control/GTP binding protein	Forward	TCCTCCCAAAGAACATCCAG	135	300	60
		Reverse	AAGTCTCCATAGCCCGGAGT			
Pp3409^2^	MFS sugar transporter	Forward	CGGATGTAGCATGGAGACTAATG	123	300	60
		Reverse	CATCTGAAATCCACCCCTTAGA			
Pp3717^2^	tripeptidyl-peptidase 1 precursor	Forward	GGCGGAGGGTTTTCAAATTA	116	300	59
		Reverse	CTTCCTGACCCATCGAACAT			
Pp3888^2^	microtubule associated protein	Forward	GGGATCAGCTCTACGTCTGC	150	300	60
		Reverse	AGATTGCCAGCAGCCTTTTA			
Pp3944^2^	autophagy-related protein 8	Forward	CCGTATTCCTGTCATCTGTGAG	107	300	60
		Reverse	CATAGACGAACTGCCCAACC			
Pp3998^2^	delta (12) fatty acid desaturase	Forward	CCCTCTCCTCCCTCACTACC	135	300	59
		Reverse	TTGGAGCACAGATGATGAGC			
Pp4000^2^	subtilase-type proteinase psp3	Forward	TCACTTTGACATTGGAACGAG	93	300	60
		Reverse	GCAACCTCAGGAAGGGTTCT			
contig2S^2^	acyl-CoA dehydrogenase	Forward	AATCGATACCGCCGTCTATG	124	300	60
		Reverse	CCTGTGTAAGCCGAAGAAGG			
contig6B^2^	conidiation-related protein	Forward	AATGACAGAGGTGGCGAGAC	107	300	60
		Reverse	TGTCGAAGCTCGTCCTTTTT			
Pp481^2^	MAS3 protein	Forward	CGTGATGGTACTCGAACGAAC	68	200	62
		Reverse	CTTGAAACCTCACGGTCTCG			
NA^3^	*P. pachyrhizi*	Forward	CCAAGGCTTCTTCGTGTTTCA	65	200	60
	α-tubulin	Reverse	CAAGAGAAGAGCGCCAAACC			

^1^Primer pairs used for quantification of mRNA transcripts throughout the infection cycle.

^2^Primer pairs used for quantification of mRNA transcripts during germination, germ tube elongation, and appressoria formation.

^3^Primer pairs used for both quantitative analyses.

### Quantitative real-time RT-PCR analysis of transcript levels during urediniospore germination, germ tube elongation, and appressoria formation

Urediniospores were germinated as described above on the surface of water for 6 and 24 h, and on an appressoria-inductive surface for 6 and 24 h. Total RNA was isolated from 100 mg of each sample and from 100 mg of urediniospores using the RNeasy Mini Plant kit (Qiagen) following the manufacturer’s protocol. Twelve target genes common to the appressoria-enriched cDNA library and the germinated urediniospore cDNA library were selected for transcript analysis, and primers were designed as described above. Quantitative real-time RT-PCR was performed on three biological replicates and three technical replicates for each target using the SmartCycler System as described above for qRT-PCR during the infection cycle. Sequences and annealing temperature for each primer set are listed in Table 1. Primers for α-tubulin were included to assess RNA integrity and demonstrate functionality of the assay [33]. For each time point, the average number of target molecules and standard deviation was calculated using the three biological replicates. Data were analysed by analysis of variance.

### Protein extraction

Appressoria-enriched samples weighing 300 mg were ground to a fine powder in liquid nitrogen and suspended in 1 ml of isoelectric focusing (IEF) buffer containing 7 M Urea, 2 M Thiourea, 4% (3-[(3-Chloramidopropyl) dimethylammonio] propanesulfonate) (CHAPS) and 25 mM dithiothreitol (DTT). The suspension was mixed thoroughly and incubated at room temperature for 30 min on a shaker at 100 rpm. Samples were centrifuged for 15 min at 14,000 g and the supernatant was collected. This protein sample will be referred to as the appressoria-enriched fraction (AEF). The AWF was collected as described above to a final volume of 500 ml per biological replicate and applied to 0.45 μm filter units (Millipore; Billerica, MA) to remove remnant urediniospores and germ tubes. Proteins were precipitated in 80% acetone at -20°C for 16 h, collected by centrifugation at 14,000 g for 20 min, and resuspended in 3 ml of resuspension buffer (10 mM Tris pH 7.0, 1 mM EDTA, 3 mM DTT, 250 μM phenylmethylsulfonyl fluroride (PMSF) and 10% glycerol (w/v)). Samples were dialyzed against 5 l of resuspension buffer for 16 h at 4°C, precipitated in acetone, and resuspended in IEF buffer. Protein quantification was performed using the Markwell assay [34] with bovine serum albumin (BSA) as a standard.

### Two-dimensional gel electrophoresis and sample preparation

Two-dimensional gel electrophoresis (2-DE) was carried out as previously described [15]. Prior to 2-DE, samples were diluted to the appropriate protein concentration in IEF buffer and 1.0% pH 3-10 carrier ampholytes (Invitrogen) were added. For first dimension 400 μg of AEF or 200 μg of AWF total protein was applied to a 13 cm pH 3-10NL Dry Gel Strip (G. E. Healthcare; Piscataway, NJ). For second dimension electrophoresis, strips were separated on 4-12% Bis-Tris gels (BioRad; Hercules, CA) and stained with Simply Blue SafeStain (Invitrogen). Gels were run from two biological replicates of AEF and 385 protein spots were picked. A total of 150 spots were picked from gels run from three biological replicates of AWF. Spots were excised from gels manually using sterile filtered pipette tips (Mettler-Toldeo; Columbus, OH). Selected protein spots were destained for 30 min at 37°C in 200 μl of a solution containing 2 mg/mL NH_4_HCO_3_ in 50% ACN/H_2_O (v/v). This step was repeated as necessary until complete removal of visible coloration. Following destaining, samples were dehydrated in 50 μl of 100% ACN solution for 15 min at room temperature. The spots were then air-dried for 10 min at room temperature, followed by trypsin-digestion for 16 h at 37°C in 20 μl of a buffer containing 20 μg/μl activated trypsin (Promega; Madison, WI), 10% ACN and 40 mM NH_4_HCO_3_ for 16 h at 37°C.

Peptides were cleaned and spotted on Matrix Assisted Laser Desorption Ionization (MALDI) plates using the Digilab/Investigator ProMS MALDI Preparation Station (Genomics Solutions; Ann Arbor, MI) programmed for peptide cleanup using the C18 ZipTip procedure according to the manufacturers recommendations (Millipore).

### Mass spectrometry

Trypsin-digested proteins were subjected to mass spectrometry analysis using a 4700 Proteomics Analyzer instrument (MALDI-Time of Flight (TOF)/TOF) (Applied Biosystems) in the positive reflectron mode as previously described [15]. Spectra between the range of 800 and 4000 Da in MS mode were acquired through the averaging of 1000 spectra, and 2000 spectra in the MS/MS mode. Up to 10 most intense ions were selected for MS/MS analysis using post-source decay (PSD) with 1 keV acceleration voltage. Criteria for ion selection were based in a signal-to-noise ratio cutoff of 20 and exclusion of all common trypsin autolysis peaks and common keratin contaminants. Instrument conversion of time-of-flight to mass (Da) for the monoisotopic ions was obtained with a tolerance of 50 ppm or better according to calibration with a peptide calibration mixture (Applied Biosystems). The MS/MS TOF calibration was optimized to 0.1 Da or better from the PSD of Glu1-fibrinopeptide B fragments.

Combined MS and MS/MS data were submitted for analysis using GPS Explorer version 3.6 software (Applied Biosystems) with MASCOT version 2.3.02 search engine (Matrix Science; Boston, MA) against a custom sequence database of EST sequences in FASTA format. The analyses criteria included the following variable modifications: methionine oxidation, formation of pyroglutamine from N-terminal glutamine, and carbamidomethylation of cysteine residues from the reduction and alkylation of proteins. Reported proteins from database searches of putative peptide/protein sequences are within ≥95% confidence interval.

The database was constructed from a subset of the NCBI EST database compiled in March 2010 using the keywords Rust or Basidiomycota. The dataset was combined with EST sequences from the appressoria-enriched SSH library described above for a total of 280,119 sequences. Identification of proteins were validated by reanalyzing all samples using a decoy database composed of randomized entries of the corresponding original database.

### Database analysis

Protein scores are based on the sum of the ion scores for the peptide mass fingerprints and MS/MS of selected peptides. A match score greater than the protein score threshold of 75 is considered significant within a 0.05 probability. The full data set from these analyses is included in the Additional file 1: Table S1.

Putative protein identities were assigned to ESTs based on BLAST searches against the NCBI nr protein database using open reading frames (ORF) corresponding to the peptides identified in each accession. BLASTX was performed using EST accessions with the highest protein score to identify protein homologues. Proteins were categorized using Uni-Prot Protein Knowledge Database [35] and placed into functional categories using the Munich Information Center for Protein Sequences (MIPS) Functional Catalogue. Proteins with multiple functions were given secondary classifications. Peptide sequences, protein BLAST identities and functional categories for each protein are listed in Additional file 1: Table S1. A comparison of Additional file 1: Table S1 to a data set of *P. pachyrhizi* germinated urediniospores proteins [15] identified a subset of different proteins for further analysis.

Proteins with putative signal peptides containing peptide cleavage sites were identified using SignalP 3.0 [36]. Target P [37] and PSORT [38] were used to predict intra- or extracellular localization of the proteins.

## Results

### Fungal strain and growth conditions

**Figure 1:**
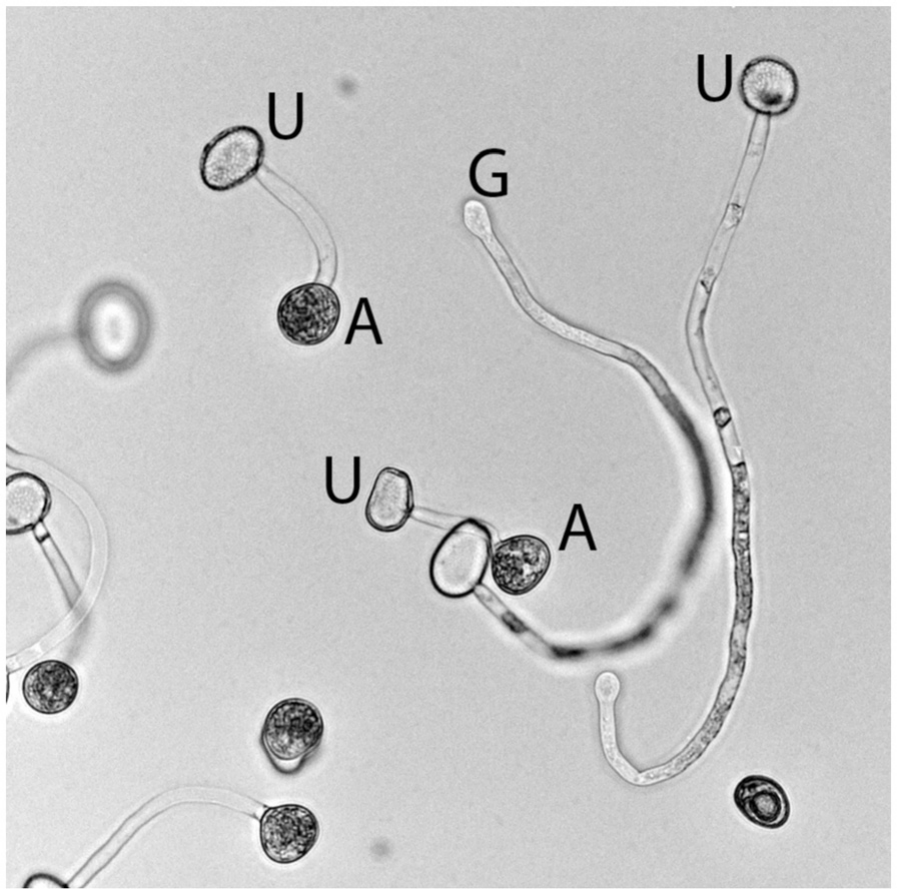
***Phakopsora pachyrhizi*appressoria.** Urediniospore (U) germination and appressoria (A) formation following 6 hour incubation on polystyrene. Apical swelling of the germ tubes is also evident (G).

After 6 h on polystyrene plates, germination rates of urediniospores averaged 60%, with 77% of germinated urediniospores showing mature, melanized appressoria (Figure 1). Washing plates with distilled water raised the percent appressoria on each plate to an average of 86%. Less than 1% of the urediniospores remaining on the plates following the wash step were ungerminated. The remainder of the urediniospores were germinated with either immature or no appressoria.

### Sequence analysis and annotation of expressed genes

**Figure 2:**
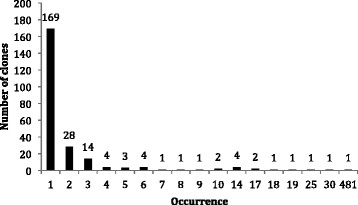
**Frequency of occurrence of ESTs derived from an appressoria-enriched cDNA library.** The number of ESTs is shown above each frequency column.

Single pass sequencing of 1133 cDNA clones led to the identification of 1029 ESTs. The sequences of the *P. pachyrhizi* EST clones were submitted to NCBI as GenBank Accession numbers JK649959 to JK650987. The remaining 104 clones sequenced contained either no inserts or consisted of poor quality sequence and were discarded. A total of 238 non-redundant ESTs were identified (Additional file 2: Table S2), of which 169 appeared only once and 69 were represented by multiple clones at frequencies ranging from 2 to 481. The frequency of redundant clones is shown in Figure 2. Assembled sequences of the redundant ESTs were submitted to NCBI as GenBank Accession numbers JR863574 to JR863646. Singleton sequences ranged from 129 to 1217 bp, and assembled contigs ranged from 424 to 1781 bp.

BLASTN analysis of single pass sequences identified 41 ESTs with no homology to *P. pachyrhizi* ESTs from germinated urediniospores and *P. pachyrhizi*-infected soybean leaves. Bi-directional sequencing was performed on these 41 ESTs, and the sequences were submitted to NCBI as GenBank Accession numbers JQ083238 to JQ083278. Of these, 29 non-redundant ESTs were identified with no homology to *P. pachyrhizi*. Twenty-seven ESTs were singletons, and two ESTs occurred twice.

Sequence similarity comparisons of the 29 non-redundant ESTs to the NCBI non-redundant protein database identified 26 ESTs, which fell into eight functional categories (Table 2). Twenty-four ESTs shared identity to proteins from members of the fungal phylum Basidiomycota, and one EST had similarity to a member of the Ascomycota. The remaining EST had identity to an IS10 transposase from tomato. Three ESTs (Pp3394, Pp3734, Pp3842) showed no significant similarity to any protein entries in GenBank.

**Table 2:** ***Phakopsora pachyrhizi*EST clones displaying similarity (BLASTX, E value <5e-05) to proteins in the NCBI non-redundant protein database, grouped into functional categories using the Munich Information Center of Protein Sequences (MIPS) Functional Catalogue**

**Clone/contig**	**Accession No.**	**Description**	**Species**	**E value**
01. Metabolism				
Pp3070	EGG05957	family 1 Carbohydrate esterase	*Melampsora larici-populina*	5.00E-9
Pp3292	EFP76188	FLU1-II (Glutamine synthetase)	*Puccinia graminis* f. sp. *tritici*	8.00e-56
Pp3391	XP_001833926	Palmitoyltransferase AKR1	*Coprinopsis cinerea okayama*	9.00E-141
Pp3684	XP_571450	Hydroxymethylglutaryl- CoA	*Cryptococcus neoformans* var.	3.00E-65
		reductase (NADPH)	*neoformans*	
Pp3741	EGG05960	family 4 carbohydrate esterase	*M. larici-populina*	8.00E-14
Pp3942	EFP86206	mannosyl phosphorylinositol	*P. graminis* f. sp*. tritici*	1.00E-128
		ceramide synthase SUR1		
10. Cell cycle and DNA processing				
Pp3291	XP_001834782	DNA replication complex	*C. cinerea okayama*	1.00E-08
		GINS protein		
Pp3495	EGD98306	G2/M phase checkpoint control	*Trichophyton tonsurans*	6.00E-26
		protein SUM2		
Pp3590	EFP88202	DNA repair protein RAD51	*P. graminis* f. sp*. tritici*	3.00E-161
Pp3976	XP_003195178	Chromatin remodeling-related	*Cryptococcus gattii*	3.00E-45
		protein		
14. Protein Fate (folding, modification, destination)				
Pp3243	XP_001838238	Prefoldin subunit 5	*C. cinerea okayama*	3.00E-26
Pp3282	XP_567169	Ubiquitin-protein ligase	*C. neoformans* var. *neoformans*	2.00E-26
20. Cellular transport, transport facilitation and transport routes				
Pp3060	XP_001883758	Cytoplasmic dynein intermediate	*Laccaria bicolor*	2.00E-128
		chain		
Pp3772	XP_571408	Membrane transporter	*C. neoformans* var. *neoformans*	3.00E-20
30. Cellular communication/signal transduction mechanism				
Pp3004	XP_571731	Serine/threonine-protein	*C. neoformans* var. *neoformans*	0.00
		kinase orb6		
Pp3435	EFP87863	Inositol hexakisphosphate kinase 2	*P. graminis* f. sp. *tritici*	8.00E-96
32. Cell Rescue, Defense and Virulence				
contig2C	XP_003192457	Heat shock transcription factor 2	*C. gattii*	1.00E-19
38. Transposable elements				
Pp3508	ABI34274	IS10 transposase, putative	*Solanum lycopersicum*	0.00
99. Unclassified				
Pp3161	EGG09840	hypothetical protein	*M. larici-populina*	6.00E-96
Pp3267	EFP87812	hypothetical protein	*P. graminis* f. sp. *tritici*	1.00E-6
Pp3464	EFP91488	hypothetical protein	*P. graminis* f. sp*. tritici*	1.00E-19
Pp3502	XP_569331	hypothetical protein	*M. larici-populina*	4.00E-115
Pp3505	XP_002396863	hypothetical protein	*M. larici-populina*	7.00E-37
Pp3853	EGG01299	hypothetical protein	*M. larici-populina*	5.00E-25
Pp3884	EFP87187	hypothetical protein	*P. graminis* f. sp. *tritici*	1.00E-24
contig2bb	EFP88656	hypothetical protein	*P. graminis* f. sp. *tritici*	3.00E-73

### Quantitative real-time RT-PCR analysis of transcripts during soybean infection

Six ESTs specific to the appressoria-enriched cDNA library were selected for analysis of transcription levels during the infection process on soybean. Transcripts were assessed via real-time RT-PCR as a measure of target molecule for each of these ESTs (genes): Pp3684 (hydroxylmethylglutaryl coenyzme A (HMG-CoA) reductase), Pp3495 (G2/M phase checkpoint control protein Sum2), Pp3243 (prefoldin subunit 5), Pp3282 (ubiquitin-protein ligase), Pp3004 (serine/threonine-protein kinase), and Pp3505 (a hypothetical protein). The sequences represented four protein functional categories and one unclassified hypothetical protein. Three ESTs common to both the appressoria-enriched cDNA library and the germinated urediniospore cDNA library were also selected for transcript analysis. These ESTs represented two protein functional categories and one unclassified hypothetical protein: contig2F (5-aminolevulinate synthase), contig2N (P-type cation-transporting ATPase), and Pp3186 (a hypothetical protein).

**Figure 3:**
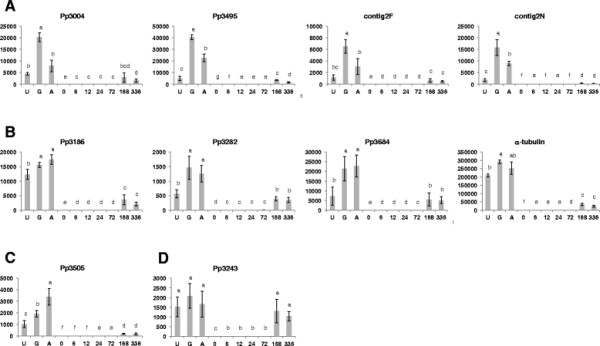
**Absolute quantification of mRNA transcripts of selected ESTs during the infection cycle.** Soybean cultivar Williams 82 was inoculated with *Phakpsora pachyrhizi* isolate Taiwan 72-1. RNA was extracted from leaves collected at 0, 6, 12, 24, 72, 168, and 336 hours post inoculation. RNA was also extracted from urediniospores (U), urediniospores germinated for 6 hours on the surface of water (G), and urediniospores germinated for 6 hours on an appressoria-inductive surface (A). The y-axis represents absolute expression of these transcripts. Error bars represent the standard deviation. Bars topped with different letters are significantly different (P < 0.05). Group **A** had highest expression in germinated urediniospores. Group **B** showed highest expression in both germinated urediniospores and appressoria, and lower expression in urediniospores. Group **C** had highest expression in appressoria and lowest expression in urediniospores.

While all of the genes were expressed in urediniospores, germinated urediniospores, and appressoria, differences were observed (Figure 3). The α-tubulin gene had the highest transcription levels. Transcripts were not detected for any of the ESTs in infected soybean leaves immediately following inoculation at 0 hpi, but transcripts were detected at subsequent time points after inoculation.

Patterns of gene expression in urediniospores, germinated urediniospores, and appressoria fell into four groups. The first group consisted of ESTs Pp3004, Pp3495, contig2F, and contig2N, with the highest transcript levels in germinated urediniospores (Figure 3A). The transcript levels in appressoria ranged from 39 to 57% that of germinated urediniospores. Transcript levels in urediniospores were 11 to 22% that in germinated urediniospores.

The second group, comprised of ESTs Pp3186, Pp3282, Pp3684, and alpha-tubulin, had high transcript levels in both germinated urediniospores and appressoria (Figure 3B). The number of target molecules in appressoria ranged from 86 to 111% that of germinated urediniospores. Transcript levels in urediniospores were less than transcript levels in both germinated urediniospores and appressoria, but the difference was less than in the previous group. The transcript levels in urediniospores ranged from 34 to 79% that of germinated urediniospores and 32 to 83% that of appressoria.

The third and fourth group were each comprised of single ESTs. Pp3505 showed highest transcription in appressoria and lowest in urediniospores (Figure 3C), while Pp3243 showed no significant difference in transcription between urediniospores, germinating urediniospores, and appressoria (Figure 3D).

### Quantitative real-time RT-PCR analysis of transcripts during urediniospore germination, germ tube elongation, and appressoria formation

**Figure 4:**
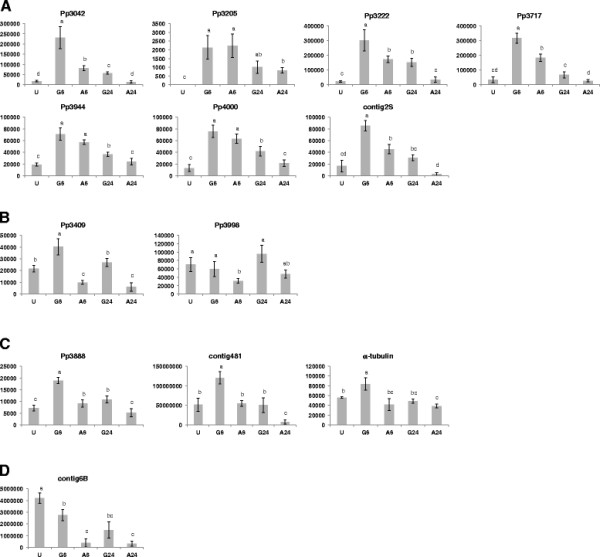
**Absolute quantification of mRNA transcripts of selected ESTs during urediniospore germination, germ tube elongation, and appressoria formation.** RNA was extracted from urediniospores (U), urediniospores germinated on the surface of water for 6 hours (G6) or 24 hours (G24), and urediniospores germinated on an appressoria-inductive surface for 6 hours (A6) or 24 hours (A24). The y-axis represents absolute expression of these transcripts. Error bars represent the standard deviation. Bars topped with different letters are significantly different (P < 0.05). Expression patterns fell into four basic groups. Group **A** had lowest expression in urediniospores and higher expression in germinated spores and appressoria at 6 hours. Group **B** showed lowest expression from appressoria at both time points. Group **C** had less variation in expression across samples. Group **D** showed the highest expression in urediniospores and lowest expression appressoria at both time points.

Twelve ESTs common to both the appressoria-enriched cDNA library and the germinated urediniospore cDNA library were selected for transcript analysis during urediniospore germination, germ tube elongation, and appressoria formation to test if genes expressed in germinated urediniospores were expressed at higher levels in appressoria. The ESTs (genes) evaluated were: Pp3042 (small GTPase-binding protein), Pp3222 (septin; cell division control/GTP binding protein), Pp3717 (tripeptidyl-peptidase 1 precursor), Pp3944 (autophagy-related protein 8), Pp4000 (subtilase-type proteinase psp3), Pp3205 (NADPH oxidase A), contig2S (acyl-CoA dehydrogenase), contig481 (*Magnaporthe* appressoria specific 3 (MAS3) protein), Pp3409 (major facilitator superfamily (MFS) sugar transporter), Pp3998 (delta 12 fatty acid desaturase), Pp3888 (microtubule associated protein), and contig6B (conidiation-related protein). All of the genes were expressed in the five samples, and the expression patterns fell into four groups (Figure 4).

The largest group consisted of the ESTs Pp3042, Pp3205, Pp3222, Pp3717, Pp3944, Pp4000, and contig2S had lowest transcript levels in urediniospores (Figure 4A). Both germinated urediniospore and appressoria samples collected at 6 h yielded high transcript levels, with germinated urediniospores tending to be higher than appressoria. The transcript levels in germinated urediniospore and appressoria samples collected at 24 h were lower than transcript levels from germinated urediniospore and appressoria samples collected at 6 h.

The second group, ESTs Pp3409 and Pp3998, had lowest transcript levels in appressoria samples collected at 6 and 24 h (Figure 4B). For Pp3409 the highest transcript level was from germinated urediniospores collected at 6 h, with the corresponding 24 h samples showing 33% fewer transcripts. Appressoria samples collected at 6 h and 24 h showed 75% and 85% fewer transcripts than germinated urediniospores at 24 h, while urediniospores showed 47% fewer transcripts. For Pp3998, the highest transcript level was from germinated urediniospores collected at 24 h, while the corresponding 6 h samples had 38% fewer transcripts. Appressoria collected at 6 h and 24 h showed 67% and 50% fewer transcripts than germinated urediniospores at 6 h. Transcript levels from urediniospores were 27% less than that found in 24 h germinated urediniospores.

The third group, consisting of ESTs Pp3888 and contig481, and α-tubulin, had less change in transcript levels across all samples than any of the other groups (Figure 4C). The highest transcript levels were observed in germinated urediniospores collected at 6 h.

The EST contig6B comprised the fourth group. The highest transcript levels were in urediniospores and lowest transcript levels occurred in appressoria (Figure 4D). Transcript levels in appressoria at 6 h and 24 h was only 9% and 7% that of urediniospores. Transcript levels in germinated urediniospores at 6 h was 66% that of urediniospores, and decreased to 35% by 24 h.

### Similarity to *Magnaporthe grisea* appressoria specific protein 3 (MAS3) and fungal proteins with predicted signal peptide sequences

**Figure 5:**
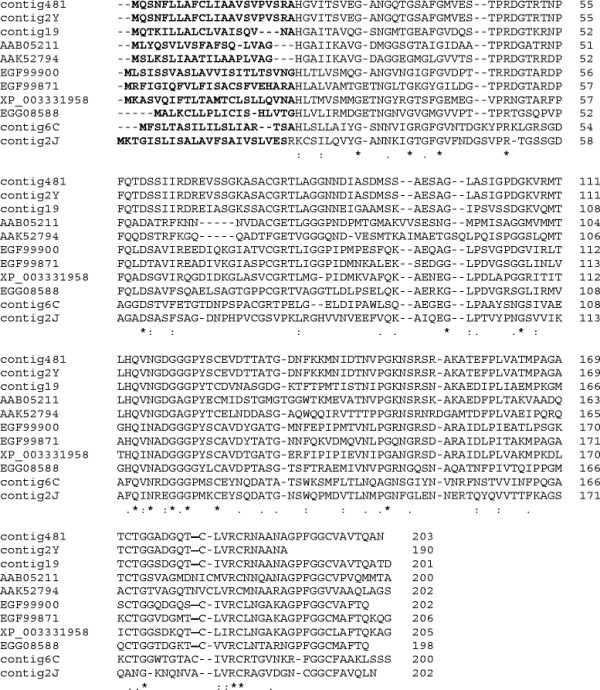
**Alignment of predicted amino acid sequences of*Phakopsora pachyrhizi*ESTs with similarity to MAS3 and other putative secreted fungal proteins.** The predicted amino acid translation of five EST contigs from the *P. pachyrhizi* appressoria-enriched cDNA library aligned with MAS3 of *Magnaporthe grisea* (AAK52794), GEgh16 from *Blumeria graminis* (AAB05211), and putative secreted proteins from *Puccinia graminis* f. sp. *tritici* (XP_003331958) and *Melampsora larici-populina* (EGF99900, EGF99871, EGG08588). Asterisks indicate amino acid identities, and dots denote conserved amino acid substitutions. Putative signal peptide sequences are in bold.

BLASTX analysis revealed five ESTs with similarity to MAS3, which was the third most redundant EST from an appressoria cDNA library of *Magnaporthe grisea*[39]. SignalP predicted all five ESTs contain putative signal peptide sequences. Contig481 had the highest identity to MAS3 at 46% (Figure 5). Among the ESTs, contig481 and contig2Y share 100% identity at the amino acid level, and contig19 has 70% identity to contig481. In addition, contig19 showed the highest identity (51%) to gEgh16 of *Blummeria graminis*. These ESTs also share identity to three secreted proteins from *Melampsora laricipopulina* and to a putative secreted protein from *Puccinia graminis* f. sp. *tritici* (Figure 5). BLASTP analysis of the open reading frames of the ESTs revealed that all have similarity to the protein superfamily DUF3129, which is a eukaryotic family of proteins with no known function.

### MALDI-TOF/TOF analysis

A total of 535 spots were excised from five 2-DE gels and analyzed in triplicate by MALDI-TOF/TOF. Data from these analyses are included as Additional file 1: Table S1. Protein identifications were validated when reanalysis of the dataset against a decoy database resulted in zero false positives. Combined MS and MS/MS analyses resulted in 480 putative identities within the established threshold limits (Additional file 1: Table S1). A total of 256 protein spots were identified in replicate gels of the AEF and AWF. Eliminating redundancy within each sample type yielded 140 proteins: 115 and 25 proteins were identified from the AEF and AWF, respectively (Additional file 3: Table S3). Four of the proteins were unique to AWF, and 55 were found only in the AEF. A total of 119 different proteins were identified in this study, 59 of which were not found in the previous analysis of proteins from germinated urediniospores of *P. pachyrhizi*[15] (Table 3). These proteins have been designated as predicted *Phakopsorapachyrhizi* proteins (PHAP).

**Table 3:** **Putative proteins identified during*Phakopsora pachyrhizi*appressoria formation grouped into functional categories using the Munich Information Center of Protein Sequences (MIPS) Functional Catalogue**

**Protein ID^1^**	**GeneInfo Identifier No.**	**Description**	**Species**	**Peptides**	**Score**
01. Metabolism					
PHAP0002	gi|254121616	2-isopropylmalate synthase	*Melampsora larici-populina*	4	151
PHAP0008	gi|120528021	5-methyltetrahydropteroyl	*Phakopsora pachyrhizi*	3	116
		triglutamate-homocysteine			
		methyltransferase			
PHAP0010	gi|120509127	acetyl-CoA C-acyltransferase	*P. pachyrhizi*	11	649
PHAP0031	gi|120522633	deoxyuridine 5'-triphosphate	*P. pachyrhizi*	6	416
		nucleotidohydrolase			
PHAP0032	gi|120523312	dienelactone hydrolase	*P. pachyrhizi*	10	243
PHAP0043	gi|120500948	glycine dehydrogenase	*P. pachyrhizi*	3	115
PHAP0064	gi|120519389	isocitrate dehydrogenase	*P. pachyrhizi*	5	102
PHAP0065	gi|120515452	isocitrate lyase	*P. pachyrhizi*	4	147
PHAP0071	gi|290897165	NADP-dependent mannitol	*M. larici-populina*	3	83
		dehydrogenase			
PHAP0103	gi|90563667	methylene-tetrahydrofolate	*Leucosporidium scottii*	3	89
		dehrogenase			
PHAP0109	gi|254151530	UTP-glucose-1-phosphate	*M. larici-populina*	7	129
		uridylyltransferase			
02. Energy					
PHAP0027	gi|120521145	cytochrome C	*P. pachyrhizi*	10	428
PHAP0089	gi|120515891	pyruvate dehydrogenase beta	*P. pachyrhizi*	12	339
PHAP0101	gi|120500717	succinate-CoA ligase beta	*P. pachyrhizi*	6	171
PHAP0107	gi|161646983	ubiquinol-cytochrome C	*Sporobolomyces roseus*	6	78
		reductase			
10. Cell cycle and DNA processing					
PHAP0024	gi|290902725	cell division cycle protein	*Melampsora medusae*	8	214
		cdc48	f. sp. *deltoidis*		
PHAP0044	gi|118187570	GTP binding protein/GTPase	*Ustilago maydis*	5	91
PHAP0074	gi|254164953	nuclear segregation protein	*M. larici-populina*	3	66
PHAP0076	gi|66651924	nucleoside diphosphate kinase	*Uromyces viciae-fabae*	5	173
PHAP0096	gi|120423194	septin	*Uromyces appendiculatus*	1	52
11. Transcription					
PHAP0084	gi|120503605	polyadenylate binding protein	*P. pachyrhizi*	9	379
12. Protein Synthesis					
PHAP0117^2^	gi|120521780	40 S ribosomal protein S16	*P. pachyrhizi*	5	89
PHAP0006	gi|282815640	40 S ribosomal protein S19	*Puccinia tricina*	10	368
PHAP0118^2^	gi|120498576	60 S acidic ribosomal protein	*P. pachyrhizi*	5	88
PHAP0009	gi|120522205	60 S ribosomal protein L10	*P. pachyrhizi*	13	732
14. Protein Fate (folding, modification, destination)					
PHAP0026	gi|169738476	cyclophilin	*U. appendiculatus*	6	118
PHAP0038^3^	gi|120515849	glucose-regulated protein	*P. pachyrhizi*	21	379
PHAP0050	gi|290908126	hsp-10 protein	*Melampsora occidentalis*	6	198
PHAP0073^3^	gi|120425527	neddylin/ubiquitin	*U. appendiculatus*	3	129
PHAP0087	gi|120523430	proteasome subunit beta	*P. pachyrhizi*	9	343
PHAP0113^3^	gi|120515648	vacuolar protease A	*P. pachyrhizi*	7	153
16. Structural & Protein Binding					
PHAP0013	gi|120527115	actin lateral binding protein	*P. pachyrhizi*	19	494
18. Regulation					
PHAP0097	gi|156597317	serine-threonine phosphatase	*Microbotryum violaceum*	3	60
PHAP0099	gi|120527960	sterol binding protein	*P. pachyrhizi*	11	113
20. Cellular transport, transport facilitation and transport routes					
PHAP0120^2^	gi|120511797	ADP, ATP carrier protein	*P. pachyrhizi*	6	96
PHAP0033	gi|120523715	electron transport flavoprotein	*P. pachyrhizi*	5	200
		alpha			
PHAP0067	gi|120512188	mitochondrial import receptor	*P. pachyrhizi*	9	423
		TOM40			
PHAP0072	gi|169735819	nascent polypeptide-associated	*U. appendiculatus*	2	109
		complex subunit alpha			
PHAP0100	gi|254113321	succinate dehydrogenase	*M. larici-populina*	8	302
32. Cell Rescue, Defense and Virulence					
PHAP0029	gi|118188717	cytochrome C peroxidase	*U. maydis*	6	195
PHAP0040	gi|120497372	glutaredoxin-1	*P. pachyrhizi*	9	323
PHAP0048	gi|120503688	heat shock HSS1	*P. pachyrhizi*	7	376
PHAP0069	gi|120509299	NADH-cytochrome b5	*P. pachyrhizi*	5	321
		reductase			
PHAP0085	gi|120514870	polyubiquitin	*P. pachyrhizi*	12	410
38. Transposable elements					
PHAP0105	gi|254151689	transposon	*M. larici-populina*	1	47
99 Unclassified					
PHAP0030	gi|120518530	cytoplasm protein	*P. pachyrhizi*	5	313
PHAP0129^2,3^	gi|120521555	hypothetical protein	*P. pachyrhizi*	3	111
PHAP0051	gi|120523258	hypothetical protein	*P. pachyrhizi*	2	191
PHAP0052^3^	gi|120510614	hypothetical protein	*P. pachyrhizi*	12	289
PHAP0093	gi|120519941	hypothetical protein	*P. pachyrhizi*	15	512
PHAP0053	gi|120534989	hypothetical protein	*P. pachyrhizi*	5	554
PHAP0054^2^	gi|103096425	hypothetical protein	*U. maydis*	3	65
PHAP0055	gi|120521631	hypothetical protein	*P. pachyrhizi*	5	172
PHAP0057	gi|120524713	hypothetical protein	*P. pachyrhizi*	5	141
PHAP0058	gi|120506400	hypothetical protein	*P. pachyrhizi*	10	190
PHAP0059^3^	gi|120508009	hypothetical protein	*P. pachyrhizi*	3	119
PHAP0060	gi|120518154	hypothetical protein	*P. pachyrhizi*	15	402
PHAP0061	gi|120519874	hypothetical protein	*P. pachyrhizi*	4	98
PHAP0062	gi|120519876	hypothetical protein	*P. pachyrhizi*	6	194

^1^Predicted *Phakopsorapachyrhizin* protei (PHAP).

^2^Proteins identified from the appressoria water fraction.

^3^Proteins containing a secretion signal.

A search of the custom EST database found that 110, or 92%, of the 119 proteins shared similarity to putative proteins from rusts. Of these 110 proteins, 90 had identity to *P. pachyrhizi* ESTs, 15 of which were from the appressoria-enriched cDNA library. The remaining 20 proteins had similarity to ESTs from *Melampsora*, *Uromyces* and *Puccinia* spp. Of the nine proteins that were not similar to any rust sequences, four had similarity to *Ustilago maydis* (cytochrome C peroxidase, GTP binding protein, a hypothetical protein, and nucleosome assembly protein), two to *Sporobolomyces roseus* (ATPase delta subunit and ubiquinol-cytochrome c reductase), one to *Leucosporidium scottii* (methylene-tetrahydrofolate dehrogenase), and two to *Microbotrium violaceum* (serine-threonine phosphatase and ubiquitin). BLASTN analysis of these nine proteins against the *P. pachyrhizi* trace archive sequences in GenBank revealed accessions with high identity to all nine proteins. The *P. pachyrhizi* genomic traces for all nine proteins shared significant amino acid similarity to *Melamspora* and *Puccinia* entries in the NCBI non-redundant protein database.

The proteins identified in this study fell into twelve functional categories (Table 3). The most abundant category of proteins at 24% was proteins with unknown function. Of the proteins with known function, proteins involved in metabolism and energy made up two of the largest group of proteins at 19% and 7%, respectively. Proteins found in these two groups include several key components of the citric acid cycle and glycolysis such as: isocitrate dehydrogenase, isocitrate lyase, acetyl-CoA C-acyltransferase, pyruvate dehydrogenase beta, and succinate-CoA ligase beta. Proteins involved in energy production included glycine dehydrogenase, NADP-dependent mannitol dehydrogenase, methylene-tetrahydrofolate dehydrogenase, and cytochrome C. An enzyme associated with glycogenesis, UTP-glucose-1-phosphate uridylyltransferase, was also identified.

Cell cycle and DNA processing accounted for 8% of proteins and included proteins involved in cell division and differentiation; specifically, cell division cycle protein cdc48, septin, and nuclear segregation protein. Protein fate accounted for 10% of proteins; cellular transport, 8%; cell rescue and defense, 8%; and protein synthesis, 7%. Regulation, transcription, protein binding, and one transposable element accounted for the remaining 9% of the proteins. Among proteins with a known function, 36% were associated with the mitochondria.

Eight proteins listed in Table 3 were predicted to contain classical secretion signals [36]. Three proteins, PHAP0038, PHAP0073, and PHAP0113, shared similarity to well-characterized intracellular proteins related to trafficking and proteolysis. PHAP0038 was identified as a glucose-regulated protein that is secreted into the ER and is involved in the assembly of protein complexes for secretion or translocation to membranes [40]. PHAP0113 was identified as a vacuolar protease A with aminopeptidase activity [41], and PHAP0073 was identified as a neddylin/ubiquitin which is required for protein assembly in the ubiquitination pathway [42].

The remaining five proteins containing a predicted secretion signal were proteins of unknown function. BLAST searches of the custom EST database identified *P. pachyrhizi* ESTs containing full-length open reading frames for four of the proteins, and a *U. maydis* EST with an ORF for the other protein. Subsequent BLASTN analysis of the *U. maydis* EST sequence identified homologous *P. pachyrhizi* genomic sequences. BLASTX analysis using *P. pachyrhizi* EST sequences corresponding to PHAP0052 and PHAP0059 found significant similarity to a *Melampsora larici-populina* nuclear membrane hypothetical protein with a conserved putative stress response domain (EGG05479.1) and hypothetical protein containing a conserved fasciclin domain (EGG10923.1), respectively. BLASTX analysis using the *P. pachyrhizi* EST corresponding to PHAP0054 revealed similarity to a conserved hypothetical protein of *U. maydis* (XP756219.1).

**Figure 6:**
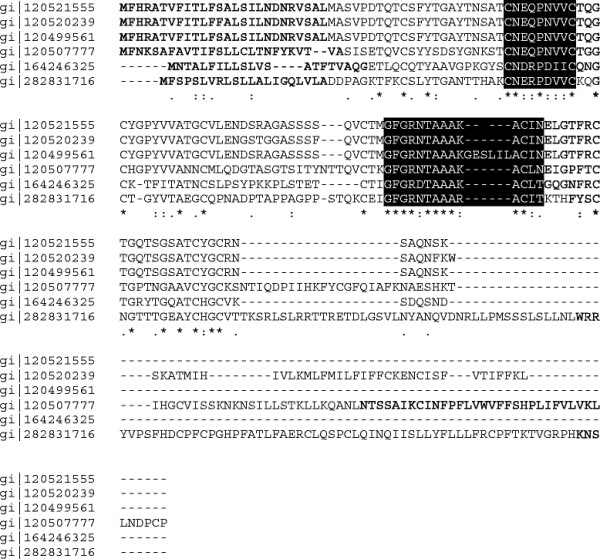
**Alignment of putative secreted proteins.** Alignment of PHAP0129, a putative secreted protein identified in the *Phakopsora pachyrhizi* appressoria water fraction, with the translated open reading frames of ESTs from *P. pachyrhizi* (gi|120499561, gi|120520239, gi|120507777), *Puccinia triticina* (gi|282831716), and *Uromyces viciae-fabae* (gi|164246325)*.* EST gi|120521555 corresponds PHAP0129. Signal peptides are indicated in bold, and conserved amino acid sequences are shaded.

Two of the *P. pachyrhizi* ESTs, gi|120521555 and gi|120521631, encode for the small molecular weight proteins PHAP0129 and PHAP0055 with 132 and 71 amino acids, respectively. Both proteins possess secretion signals with no motifs for localization to organelles, suggesting they may function as extracellular fungal effectors [43]. EST gi|120521631 did not share similarity to any DNA or amino acid sequences currently in GenBank and is a unique *P. pachyrhizi* extracellular protein (PHAP0055). BLASTX analysis using the EST for PHAP0129 identified sequence similarity to ESTs from *Puccinia triticina* (gi|282831716) and *Uromyces viciae-fabae* (gi|164246325). The translated ORFs of three *P. pachyrhizi* ESTs (gi|120499561, gi|120520239, gi|120507777) were also found to share significant amino acid similarity to PHAP0129. Alignment of the predicted proteins of these six ESTs revealed two conserved regions (Figure 6).

## Discussion

This study identified ESTs and proteins present during, and possibly required for, appressoria formation in *P. pachyrizi*. Spore germination, germ tube elongation, and appressoria formation are all part of a single complex physiological process from which individual steps cannot be readily uncoupled. Therefore, the collection of data specific to appressoria formation presents a technical challenge. Suppression subtractive hybridization (SSH) was utilized to generate a cDNA library enriched for transcripts specifically involved in appressoria formation, while reducing the occurrence of transcripts also associated with urediniospore germination and germ tube elongation. Generation of a cDNA library also allowed for the possible detection of transcripts that may be present in relatively low copy number. Alternatively, 2-DE and MS analysis of proteins extracted from appressoria offered a profile of high abundance proteins, which may not be reflective of corresponding transcript levels. Combining these two techniques provided a clearer image of the molecular and biochemical processes occurring during germination, germ tube elongation, and appressoria formation than either method viewed alone.

Comparison of the appressoria-enriched cDNA library to existing ESTs from germinated urediniospores of *P. pachyrhizi* revealed 29 non-redundant ESTs specific to the appressoria-enriched cDNA library. Ten of the 29 transcripts (35%) have roles in metabolism or cell cycling. This is to be expected given the importance of both autophagy and mitosis in appressoria formation. Autophagy is widely conserved among eukaryotes and is responsible for the degradation and recycling of proteins, organelles and cytoplasm in response to stress conditions that allow the cell to adapt to environmental or developmental changes [44]. In the absence of exogenous nutrients before host colonization, spore germination, germ tube elongation and appressoria formation must be fuelled by the breakdown of spore contents. Additionally, the metabolism of lipids, glycogen, and sugars from the spore are utilized for the biosynthesis of glycerol in the appressorium, thereby generating the necessary turgor pressure to allow for penetration of the host cuticle. This breakdown and recycling of old cellular components has been shown to play a key a role in pathogenicity of fungal pathogens, including *M. grisea* (*M. oryzae*), *Colletotrichum orbiculare*, and *Fusarium graminearum*[45-47]. The *P. pachyrhizi* appressoria-enriched ESTs (Table 2) include genes that encode proteins involved in fungal metabolism and autophagy, such as HMG-CoA reductase, a key enzyme for cholesterol biosynthesis, and glutamine synthetase, an important enzyme in amino acid metabolism. Expression analysis of HMG-CoA reductase indicates that this transcript is present in high levels in both germinated urediniospores and appressoria, but in relative low levels in urediniospores prior to germination (Figure 3, EST Pp3684). HMG-CoA reductase has also been shown to be up-regulated in appressoria of *M. grisea*[48]. Similarly, glutamine synthetase is highly expressed during pathogenesis of *Colletotrichum gloeosporiioides* on *Stylosanthes guianensis*[49].

Cell cycling is pivotal for multicellular eukaryotes to coordinate the differentiation of tissues and organs. Appressoria formation requires switching from polarized hyphal growth, to expansion and cellular differentiation of appressoria, to ultimate resumption of polarized growth during penetration peg formation. Entry into mitosis, specifically the S phase, is required to regulate initiation of appressoria morphogenesis and conidial cell death in *M. grisea*[45]. Blockage of cell cycling at later stages of mitosis did not affect appressoria formation, suggesting that the checkpoint regulating cellular differentiation operates at the G2-M boundary [50]. A transcript for the G2/M phase checkpoint control protein SUM2 was identified among the *P. pachyrhizi* appressoria-enriched ESTs (Table 2). Interestingly, qRT-PCR analysis showed transcript levels in germinated urediniospores to be nearly twice that in appressoria. However, transcript levels in appressoria were more than four times that in urediniospores prior to germination (Figure 3, EST Pp3495).

Cyclic AMP-, MAP kinase-, and calcium/calmodulin-dependent signaling pathways are involved in the induction and development of appressoria [51-53]. In *P. pachyrhizi*, a putative serine/threonine protein kinase was identified and expressed in germinated urediniospores and appressoria (Figure 3, EST Pp3004). Serine/threonine kinases play a role in autophagy and fungal morphogenesis. Autophagy is blocked in mutants of the MgATG1 gene in *M. grisea*, resulting in reduced lipid turnover, inadequate appressorial turgor, reduced ability to penetrate and infect a host, and decreased conidiation [54].

An EST from the *P. pachyrhizi* appressoria-enriched cDNA library was found with 98% similarity to autophagy-related protein 8 (Atg8) from *Moniliophthora perniciosa* (GenBank accession ACD93204) [55]. During autophagy, membrane-bound autophagosomes are formed, delivered to and fused with lysosomes or vacuoles where their contents are degraded. Atg8 is one of two ubiquitin-like proteins required for autophagosome formation [56]. Targeted mutation of Atg8 in *M. grisea* arrested conidial cell death via autophagy and prevented production of penetration hyphae, thus, preventing appressoria-mediated penetration of the host cuticle [45]. The role of Atg8 during germination and appressoria formation in *P. pachyrhizi* was supported by qRT-PCR analysis, which found increased transcript levels in germinated urediniospores and appressoria relative to urediniospores (Figure 4, EST Pp3944).

BLASTX analysis of appressoria-enriched ESTs revealed eight with similarity to hypothetical proteins of unknown function from *P. graminis* f. sp. *tritici* or *M. laricis-populina*. These two fungi, along with *P. pachyrhizi,* are members of the Order Puccinales*.* ESTs Pp3502 and contig2bb also showed similarity to other members of the Basidiomycota, while the other six ESTs may represent rust-specific transcripts. Gene expression analysis of EST Pp3505 revealed the highest transcript levels occur in appressoria relative to urediniospores and germinated urediniospores (Figure 3., EST Pp3505), supporting a role for this transcript in appressoria formation of *P. pachyrhizi.* BLASTX analysis of three additional ESTs, Pp3394, Pp3734, and Pp3842, did not find any significant similarity to any entries in the NBCI non-redundant protein database, suggesting that these transcripts represent genes specific to *P. pachyrhizi*.

Of the 238 non-redundant ESTs identified, 209 (88%) were found in common with previously sequenced ESTs from germinated urediniospores and infected soybean leaves. It is reasonable to expect that a portion of the genes involved in the cascade of events required for appressoria morphogenesis may be triggered early during spore germination and germ tube elongation. Additionally, some of the genes required for appressoria formation may be the same genes necessary for spore germination and germ tube elongation. Identification of genes known to play a role in appressoria formation, penetration peg formation, and early infection in both the appressoria-enriched ESTs and the germinated urediniospore ESTs reinforces this expectation. Analysis of transcripts in other plant pathogenic fungi have shown greater changes in gene expression occur during spore germination, with fewer changes subsequently during germ tube elongation and appressoria formation [48,57].

A putative NADPH oxidase was identified among the ESTs common to both *P. pachyrhizi* cDNA libraries. NADPH oxidases generate reactive oxygen species (ROS) that are involved in various physiological processes and cellular differentiation in fungi [58]. Deletions of NADPH oxidase (*Nox*) genes block differentiation of sexual fruiting bodies in *Aspergillus nidulans*[59] and differentiation of ascogonia to perithecia in *Podospora anserins*[60]. In *M. grisea Nox1* and *Nox2* mutants formed normal looking appressoria but failed to penetrate the host, suggesting a role for Nox-derived ROS in penetration peg formation [61]. In *P. pachyrhizi*, qRT-PCR analysis revealed little to no expression of NOX in urediniospores, while expression in both germinated urediniospores and appressoria were equally high at 6 h, and reduced by more than half at 24 h. This profile matches the proposed role of ROS as a signalling pathway in cellular differentiation. Such signalling is unnecessary in dormant spores, but in high demand during germination and appressoria formation. It has been shown in *M. grisea* that the generation of ROS occurs during conidial germination, appressoria development, and during hyphal tip growth [61].

Another EST common to both *P. pachyrhizi* EST libraries, was a putative subtilase-type proteinase. The transcript levels of a subtilase-type proteinase in appressoria is nearly five times that observed in urediniospores, and the expression levels in germinated urediniospores is nearly six times that of urediniospores. Subtilisin-like serine proteases were abundant in both a cDNA library and a serial analysis of gene expression (SAGE) of appressoria of *M. grisea*[62,63]. Targeted deletion of the vacuolar serine protese, SPM1, resulted in decreased sporulation and appressoria development, and attenuated infection [64].

P-type ATPases are integral membrane proteins required for the maintenance of phospholipid asymmetry in biological membranes, and they are important for infection-related morphogenesis, such as penetration peg formation [65]. P-type ATPase ESTs were identified in both *P. pachryhizi* cDNA libraries. The penetration defective mutant, PDE1, of *M. grisea* exhibited reduced appressoria-mediated penetration and reduced disease symptoms on a susceptible host [65]. They may also be significant for the delivery of virulence-associated proteins. A mutation of MgApt2 in *M. grisea* inhibited the hypersensitive response in resistant rice cultivars, suggesting that the secretion of fungal proteins perceived by the host during a resistance response might also require MgApt2 [66]. The qRT-PCR analysis of the P-type ATPase in *P. pachyrhizi* showed increased transcript levels in germinated urediniospores and appressoria, compared with low levels in urediniospores, suggesting a possible role in germination and appressoria formation.

Expression levels of genes analyzed by qRT-PCR found most transcripts to be present in urediniospores, usually at low levels relative to germinated urediniospores and appressoria (Figure 3 and Figure 4). The presence of most transcripts at low levels in dormant spores indicates that stabilized transcripts may be stored in the spore for translation during, or immediately following, germination [67]. Two *P. pachyrhizi* transcripts did not fit this profile. Transcript levels of EST Pp3205 (NADPH oxidase) in urediniospores were negative in two of three replicate runs. Contig6B had high transcript levels in urediniospores, with decreased levels in germinated urediniospores, and low levels in appressoria. This transcription pattern is consistent with contig6B’s putative identification as a conidiation-related protein 6 (CON6). The gene *con-6* is expressed during conidiation in *Neuropsora crassa*, but is not expressed in mycelium. It was shown that shortly after spore germination *con-6* mRNA disappears and the CON6 polypeptide is rapidly degraded [68].

Of the 119 proteins identified from appressoria-enriched preparations, 59 (49.6%) proteins were not identified in the previous study of proteins from germinated urediniospores [15]. The differential protein profiles may be indicative of metabolic and physiological differences between germination on water versus a solid substrate. Differential expression was not as great between the two cDNA libraries as it was between the two proteomes, suggesting a possible greater influence of surface contact on translation than on transcription. As with the ESTs, the majority of proteins with an identified putative function play a role in metabolism. Additionally, proteins involved in cell cycling, protein fate, and cellular transport are well represented. Several of these, neddylin/ubquitin, septin, GTP binding protein/GTPase are discussed below for their potential role in appressoria formation and early infection.

Isocitrate lyase was one of the proteins identified in this study, and is necessary for the utilization of fatty acids and is required for pathogenicity in *Leptosphaeria maculansM. grisea,* and *Colletotrichum lagenarium*[69-71]. Isocitrate lyase is highly expressed in *M. grisea* during conidial germination, appressoria formation, and penetration peg formation, indicating that the glyoxylate cycle is stimulated at this time. Lipid metabolism is likely important for turgor generation in appressoria via the synthesis of glycerol.

Fifteen proteins identified in the appressoria-enriched preparations were also found as ESTs in the appressoria-enriched cDNA library. All fifteen of these represented common, well-characterized proteins such as actin, catalase, α-tubulin, and aldehyde reductase, and all were also present in both the germinated urediniospore cDNA library and the partial proteome of germinated urediniospores. It is possible that during germination and germ tube elongation, transcripts are accumulating without activation of subsequent translation. Alternatively, transcripts may be translated, but the protein turnover rate may be such that it precludes identification by 2-DE. The nature of the two techniques used may also explain some of the variation. Generation of a SSH cDNA library allows for the detection of transcripts that may be present in relatively low copy number, while 2-DE detects proteins of relative high abundance.

While 59 proteins from the appressoria-enriched preparations did not share any sequence similarity with the 29 appressoria-enriched ESTs, commonality of function was identified. Neddylin/ubiquitin was identified among the proteins (Table 3, PHAP0073), while ubiquitin-protein ligase was found among the ESTs (Table 2, EST Pp3282). Both of these proteins are involved in the ubiquination of proteins targeted for degradation in the proteasome. In conjunction with E1 (ubiquitin-activating enzyme) and E2 (ubiquitin-conjugating enzyme), neddylin acts as an intermediate step through a covalent bond with the target protein and forms a bridge between E2 and E3 (ubiquitin-protein ligase) [42]. Expression levels of the ubiquitin-protein ligase in appressoria and germinated urediniospores were more than double that of urediniospores (Figure 3, EST Pp3282).

A GTP binding protein (GTPase) was identified among the proteins (Table 3, PHAP0044) and ESTs (Table 1, EST Pp3042). Transcript analysis by qRT-PCR indicates that EST Pp3042 was highest during urediniospore germination and early germ tube elongation (Figure 4). GTP binding proteins have been shown to affect the formation of septa in infectious hyphae of *U. maydis*[72]. Septa are critical for appressoria formation and likely function as mechanical support for generation of turgor pressure necessary to differentiate appressoria and mediate mechanical penetration of the host cuticle [72]. Transcript levels of a putative septin were elevated in germinated urediniospores and appressoria at 6 hpi relative to urediniospores (Figure 4, EST Pp3222). Septin was also identified among the appressoria proteins (Table 3). Septins are conserved cytoskeletal GTPases with multiple functions, including organizational markers during cell division and polarized growth. In filamentous fungi, septins assemble into a wide variety of complexes, including those that form at growing hyphal tips and at the site of future septum formation. In *M. oryzae* deposition of the septin ring defines the position of the appressorium septum prior to mitosis in the germ tube, and septin ring formation appears to be regulated by the DNA replication checkpoint that initiates appressorium morphogenesis [73]. In *U. maydis* septin mutants have reduced symptom development on maize and produce fewer mature teliospores than wild-type [74], which is consistent with the requirement for septin during morphogenesis and cellular division.

A putative acyl-CoA dehydrogenase EST was found in both germinated urediniospore and appressoria cDNA libraries (Figure 4, EST contig2S), and acetyl-CoA acyltransferase was identified among the high abundance proteins (Table 3). These two enzymes are involved in the first and last steps of fatty acid β-oxidation, which is essential for the development and melanization of appressoria in *M*. *grisea*[75].

Five non-redundant EST contigs were identified with partial homology to one another and shared similarity to MAS3 of *M. grisea* and gEgh16 of *Blumeria graminis* f. sp. *hordei*. MAS3 and gEgh16 are both members of multigene families and are expressed during early infection [39,76,77]. Similarly, the five contigs may comprise a multigene family in *P. pachyrhizi*. Expression analysis of the most redundant transcript, contig481, revealed that this putative MAS3 homolog was highly expressed in germinated urediniospores and present in urediniospores at levels similar to those in appressoria (Figure 4, EST contig481).

Prior to removing redundancy, nearly half (49.6%) of the clones sequenced from the appressoria-enriched cDNA library showed similarity to MAS3 and gEgh16. The most abundant ESTs, assembled into contig481, showed 99% nucleotide similarity to the most abundant EST from the *P. pachyrhizi* germinated urediniospore cDNA library [14]. However, MAS3 was not identified within the partial proteomes of appressoria or germinated urediniospores [15]. Because 2-DE selectively yields high abundance proteins, the absence of MAS3 indicates that the protein is either absent or present at low levels during spore germination, germ tube elongation, and appressoria formation.

Deletion mutants of MAS3 in *M. grisea* were normal in germination, germ tube elongation, and appressoria formation, but were defective in penetration of the host and showed reduced virulence [39]. This suggests that the role of MAS3 arises post-appressoria formation. While transcripts are present, it is not until the infection process reaches the stage of penetration peg formation or actual penetration that MAS3 plays a role during infection. There may be a signal from the mature appressoria or the host plant that triggers translation at the critical point between appressoria formation and penetration.

Eight putative extracellular proteins were identified in AEF and AWF (Table 3). Three of these are well-characterized intracellular proteins that share similarity to proteins in other fungi: glucose-regulated protein, neddylin/ubiquitin, and vacuolar protease A. Four proteins share similarity to hypothetical proteins found in other fungi. The remaining putative extracellular protein, PHAP0055, did not have any similarity to proteins in the NCBI non-redundant protein database, suggesting it is unique to *P*. *pachyrhizi*.

The *P. pachyrhizi* protein PHAP0059 contains a conserved fasciclin domain which has been demonstrated to be involved in cell adhesion, appressoria turgor, and pathogenicity in *M. oryzae*[78,79]. Interestingly PHAP0059 was only found in the AEF and not among the germinated urediniospore proteins. The AEF was generated on a hard surface, while urediniospores were germinated by floating on the surface of water. This suggests that contact with a hard surface may be necessary to induce signals that regulate transcription or protein processing for translation of cell adhesion proteins.

Two *P. pachyrhizi* proteins, PHAP0055 and PHAP0129, exhibit characteristics similar to those of putative effector proteins identified from a haustoria-enriched proteome of *P. triticina*[43]. Both are small molecular weight proteins, with no known function or conserved domains, and contain a predicted signal peptide cleavage site at the N-terminus. PHAP0129 also contains an even number of cysteine residues which is characteristic of some plant-targeted fungal effector proteins [21]. In addition, PHAP0129 was found only in the AWF, further supporting its putative identification as an extracellular protein. Proteins with high identity to PHAP0129 were found in *P. pachyrhizi, U. viciae-fabae* and *P. triticina,* suggesting a putative effector protein family exists in rusts.

## Conclusions

This study utilized SSH and 2-DE and MALDI-TOF/TOF to identify genes and proteins expressed during appressoria formation. Genes and proteins involved in early infection by *P. pachyrhizi* are candidates for targeted control measures. Several genes and proteins were identified as unique to *P. pachyrhizi* and/or other rusts. One of these unique proteins is a putative extracellular effector protein. A potential rust-specific effector protein family was also identified. Determining the role of these effector proteins will be an important step in understanding the mechanisms of pathogenesis.

## Competing interests

The authors declare that they have no competing interests.

## Authors’ contributions

CLS participated in the design and execution of the experiments. She performed the soybean inoculations, developed the method for generating appressoria on polystyrene plates, extracted RNA, analyzed ESTs, performed qRT-PCR, and drafted the manuscript. MBM conducted the protein extractions and 2-DE, analyzed the MALDI-TOF/TOF data, and assisted in preparing the manuscript. LLF constructed the custom EST database, conducted the MALDI-TOF/TOF, and assisted in the proteomic analysis. AN oversaw the MALDI-TOF/TOF and assisted in the proteomic analysis. GWS developed computer algorithms for EST analysis. DGL and RDF conceived of the project, wrote the plan for implementation, oversaw the execution of the experiments and data analysis, and revised and edited the manuscript. All authors read and approved the final manuscript.

## Supplementary Material

Additional file 1 **Table S1.** Proteins identified using the custom EST database. Click here for file

Additional file 2 **Table S2.** Non-redundant *Phakopsora pachyrhizi* clones identified from the appressoria-enriched cDNA library. Click here for file

Additional file 3 **Table S3.** Non-redundant proteins identified using the custom EST database. Click here for file
